# Automated Estimation of Mammary Gland Content Ratio Using Regression Deep Convolutional Neural Network and the Effectiveness in Clinical Practice as Explainable Artificial Intelligence

**DOI:** 10.3390/cancers15102794

**Published:** 2023-05-17

**Authors:** Chiharu Kai, Sachi Ishizuka, Tsunehiro Otsuka, Miyako Nara, Satoshi Kondo, Hitoshi Futamura, Naoki Kodama, Satoshi Kasai

**Affiliations:** 1 Department of Radiological Technology, Faculty of Medical Technology, Niigata University of Health and Welfare, Niigata City 950-3198, Niigata, Japan; chiharu-kai@nuhw.ac.jp (C.K.); kodama@nuhw.ac.jp (N.K.); 2 Otsuka Breastcare Clinic, Tokyo 121-0813, Japan; 3 Department of Breast Surgery, Tokyo Metropolitan Cancer and Infectious Disease Center, Komagome Hospital, Tokyo 113-8677, Japan; 4 Graduate School of Engineering, Muroran Institute of Technology, Muroran City 050-8585, Hokkaido, Japan; 5 Konica Minolta, Inc., Tokyo 100-0005, Japan

**Keywords:** mammogram, breast composition, mammary gland content ratio, regression deep convolutional neural network, explainable AI

## Abstract

**Simple Summary:**

In Japan, a guideline for breast types, called “breast composition,” was recently developed based on BI-RADS. The Japanese guidelines are characterized using a continuous value called the mammary gland content ratio, calculated using the density of the pectoralis muscle as an indicator to determine breast composition. Discriminative DCNN has been developed conventionally to classify breast composition; however, it could encounter two-step errors or more (e.g., estimating “Fatty” as “Heterogeneous dense”). We developed a regression DCNN based on the mammary gland content ratio defined in the Japanese guideline to solve the above problem, followed by automated breast composition classification based on the continuous value. We also examined the usefulness of the continuous value of the mammary gland content ratio.

**Abstract:**

Recently, breast types were categorized into four types based on the Breast Imaging Reporting and Data System (BI-RADS) atlas, and evaluating them is vital in clinical practice. A Japanese guideline, called breast composition, was developed for the breast types based on BI-RADS. The guideline is characterized using a continuous value called the mammary gland content ratio calculated to determine the breast composition, therefore allowing a more objective and visual evaluation. Although a discriminative deep convolutional neural network (DCNN) has been developed conventionally to classify the breast composition, it could encounter two-step errors or more. Hence, we propose an alternative regression DCNN based on mammary gland content ratio. We used 1476 images, evaluated by an expert physician. Our regression DCNN contained four convolution layers and three fully connected layers. Consequently, we obtained a high correlation of 0.93 (*p* < 0.01). Furthermore, to scrutinize the effectiveness of the regression DCNN, we categorized breast composition using the estimated ratio obtained by the regression DCNN. The agreement rates are high at 84.8%, suggesting that the breast composition can be calculated using regression DCNN with high accuracy. Moreover, the occurrence of two-step errors or more is unlikely, and the proposed method can intuitively understand the estimated results.

## 1. Introduction

The number of breast cancer cases has been increasing yearly, and one in nine Japanese women is reported to have breast cancer [1]. The five-year survival rate for breast cancer is high, and early cancer detection leads to a higher cure rate. Hence, it is crucial to have breast cancer screening to detect cancer early and start treatment before subjective symptoms appear. Mammography, which is also recommended by the government for the screening of breast cancer, is the only proven test to reduce mortality [2,3,4,5]. However, abnormal lesions may be hidden by mammary tissues because both are shown as bright areas in mammograms, so the sensitivity of lesion detection depends on the amount of mammary tissue [6,7].

Recently, breast types are categorized into four types based on the amount and distribution of mammary and fatty tissues. The American College of Radiology (ACR) developed a guideline, “The Breast Imaging Reporting and Data System (BI-RADS) atlas” for breast types: (a) the breasts are almost entirely fatty; (b) there are scattered areas of fibroglandular density; (c) the breasts are heterogeneously dense, which may obscure small masses; (d) the breasts are extremely dense, which lowers the sensitivity of mammography. A dense breast, which includes the higher density types (c) and (d) [8], has a high probability of decreased sensitivity and makes lesion detection difficult. It has also been reported that a dense breast is associated with an increased risk of breast cancer [9,10,11,12,13,14,15,16,17,18,19]. In March 2023, the U.S. Food and Drug Administration mandated notification of breast types to patients [20], so it is increasing demand and vital to evaluate breast types in medical screening and daily practice.

**Figure 1:**
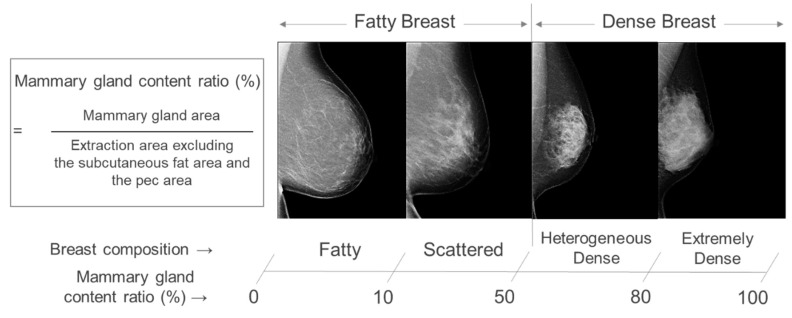
Guideline on breast composition developed by The Japan Central Organization on Quality Assurance of Breast Cancer Screening.

The Japan Central Organization on Quality Assurance of Breast Cancer Screening developed a guideline for breast types: “Fatty,” “Scattered,” “Heterogeneous dense,” and “Extremely dense” based on BI-RADS [21]. In Japan, the concept of these breast types is called “breast composition.” The guideline defines mammary gland content ratio as the area of the mammary gland equal to or greater than the density of the pectoralis muscle divided by the area in the breast where mammary tissues are thought to be present. Mammary gland content ratio less than 10% is defined as “Fatty,” 10% to less than 50% as “Scattered,” 50% to less than 80% as “Heterogeneous dense,” and 80% or more as “Extremely dense” (Figure 1). Moreover, as with BI-RADS, “Fatty” and “Scattered” are grouped as “Fatty breast,” and “Heterogeneous dense” and “Extremely dense” are grouped as “Dense breast.” This method uses the density of the pectoralis muscle as an indicator to objectively calculate the percentage of the mammary gland content ratio. This allows for a more objective evaluation as well as a visual one, while the Japanese guidelines are based on BI-RADS.

Breast composition has received considerable interest, so automated quantitative classification of breast composition has been developed using deep learning in recent years [22,23,24,25,26]. Particularly, the discriminative deep convolutional neural network (DCNN) model is standard. Gastounioti et al. [22] reported a review of automated estimation of breast types as part of Artificial Intelligence (AI) study using mammograms. Wu et al. [23] developed a high-accuracy automated classification (four categories, Cohen’s kappa = 0.61) using over 200,000 examinations. Lehman et al. [24] developed a high-accuracy automated classification (four categories, Cohen’s kappa = 0.67) using ResNet-18. Chang et al. [25] conducted a large cohort study with 92 physician’s evaluations on a 33-center imaging dataset and developed a high-accuracy automated classification (four categories, Cohen’s kappa = 0.67). Deng et al. [26] developed a high-accuracy automated classification (four categories, agreement rate = 92.17%) using a general convolutional neural network model and SE-Attention mechanism. All these studies achieved high-accuracy automated classification using discriminative DCNNs; however, it is possible to encounter two-step errors or more. An example of “two-step errors or more” is when a Fatty classification is wrongly classified as Heterogeneous dense or Extremely dense. In the studies of Lehman et al., Chang et al., and Deng et al., there were two-step errors or more [24,25,26]. Two-step errors or more are fatal because they are unlikely to occur in physicians’ evaluation and can cause physicians to be less convinced to use AI.

Therefore, we focused on the fact that the mammary gland content ratio defined in the Japanese guideline is a continuous value. We considered that high-accuracy estimation of the mammary gland content ratio using regression DCNN and the determination of thresholds to classify breasts into four categories would enable us to estimate breast composition with a lower chance of two-step errors or more. We aimed to develop an AI model for high-accuracy estimation of the mammary gland content ratio using regression DCNN and a high-accuracy automated classification of breast composition based on a continuous value from the estimated ratio to solve the above problem. We also examined the usefulness of the continuous value of the mammary gland content ratio.

## 2. Materials and Methods

**Figure 2:**
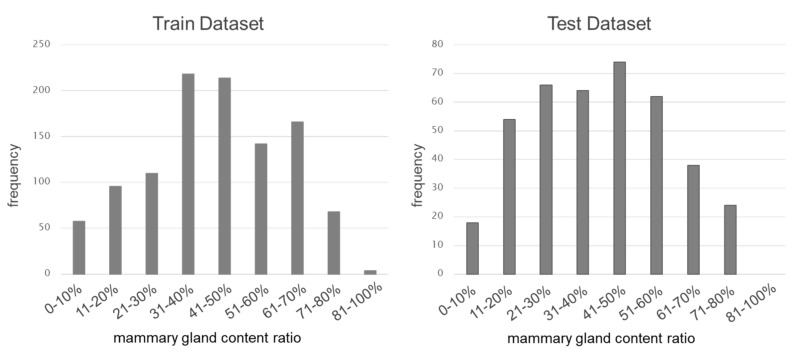
Distribution of mammary gland content ratio (training and testing datasets).

We used the guideline-recommended medio-lateral-oblique (MLO) view of the mammograms to evaluate breast composition. The images were obtained from three sites. The images selected for use were judged by the collection site physician to be normal cases and by the radiologists to have good quality positioning in all images collected from each facility over a specified period. Consequently, a total of 1476 images were selected: 828, 96, and 552 images captured using Canon FPD, Konica Minolta, and Siemens FPD, respectively. Collection periods are listed in Table 1. The 1476 images were evaluated by an expert physician who has extensive experience and is one of the main members of the guideline development team. There are two images per one subject, because we used both the left and right MLO images of the mammogram. During the evaluation, images were displayed on a high-resolution monitor (JVC LCD Monitor (5 MP), JVCKENWOOD Corporation, Tokyo), and the room was darkened as in a typical environment. The physician evaluated the breast composition (“Fatty,” “Scattered,” “Heterogeneous dense,” and “Extremely dense”) and mammary gland content ratio (0–100%). The physician also re-evaluated the images for abnormal findings and good quality positioning. Consequently, the physicians judged all 1476 images to be suitable for AI development. The images were divided into 1076 training and 400 testing images randomly. The breakdown of the breast composition by the physician is shown in Table 1. The mammary gland content ratio distribution determined by the physician in the training and testing datasets is shown in Figure 2. The dataset used had a distribution similar to the Japanese breast composition, with a high number of scattered and heterogeneous dense and a low number of fatty and extremely dense [27], confirming the validity of the database. The matrix size/pixel spacing of the images were 2016 × 2816/0.082 mm for Canon FPD, 4040 × 5416/0.04375 mm for Konica Minolta CR, and 2082 × 2800/0.085 mm for Siemens FPD. All images were unified in the left-MLO view, and the image size was changed to 8-bit grayscale and a matrix size of 202 × 282.

The mammograms used in this study were collected by Konica Minolta and shared as anonymously processed information because they did not contain personal information. However, Konica Minolta did not have any role in study design, analysis, model development, or manuscript preparation. The Institutional Review Board of Niigata University of Health and Welfare approved this study (Approval No. 18884-220829).

**Figure 3:**
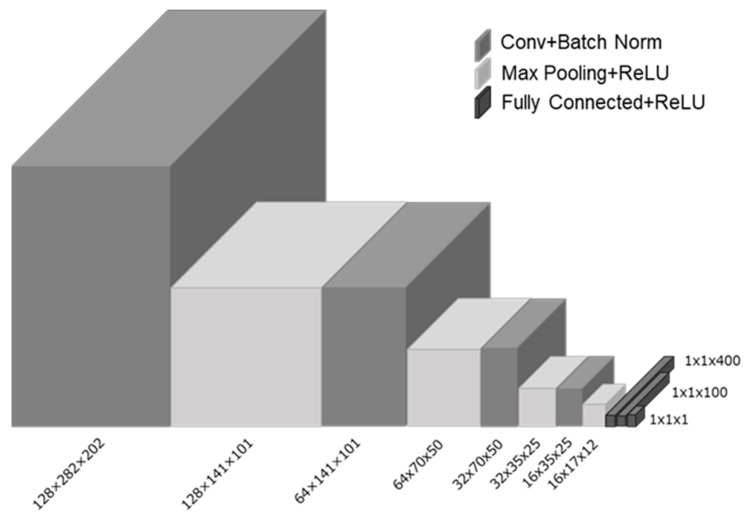
Regression DCNN model of the highest correlation coefficient with four convolution layers and three fully connected layers.

We developed a method to estimate the mammary gland content ratio using regression DCNN. We used a framework for no-code development of deep learning and the neural network console [28] (Sony Corporation, Tokyo) and varied the architecture of the regression DCNN by modifying the convolution layers and parameters of the kernel. Furthermore, we implemented data augmentation using rotation angles from 30 to 180°, in 30° increments. The other learning parameters included three fully connected layers: Rectified Linear Unit (ReLU) for the activator function, squared error for the loss function, and batch normalization. The learning rate was 0.001, and the batch size was 64. We examined various architectures of the regression DCNN and compared the correlation coefficient between the evaluation of the physician and the estimated mammary gland content ratio. The regression DCNN model with the highest correlation coefficient is shown in Figure 3. It has four convolution layers (number of channels are 128, 64, 32, and 16, respectively) and 60° rotation at maximum in data augmentation. We used a computer with a Ryzen 7 5800X CPU, 64 GB of main memory, and an NVIDIA GeForce RTX 3090 GPU (NVIDIA Corporation, Santa Clara, California, U.S.A.) for processing. Additionally, we searched thresholds of mammary gland content ratio to classify as fatty, scattered, heterogeneous dense, and extremely dense. Thresholds were determined to ensure the highest agreement rate compared with the evaluation of breast composition by the physician. For statistical analyses, we used the vcd package in RStudio (version 1.1.456) to calculate a weighted Cohen’s kappa value and 95% confidence interval calculation.

## 3. Results

**Figure 4:**
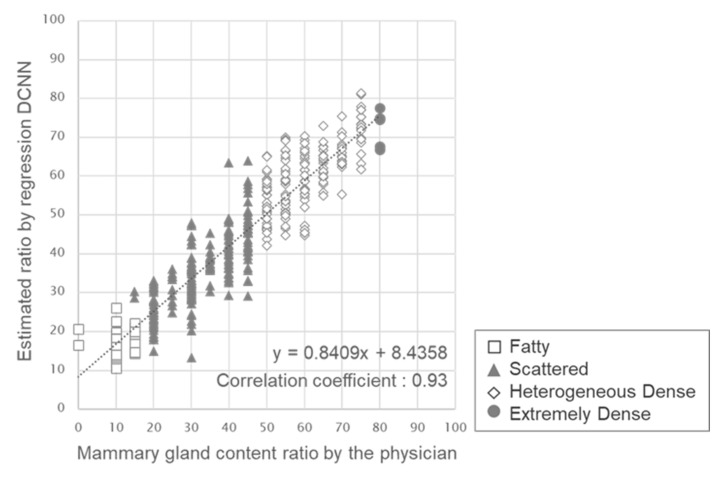
Result of the correlation between the physician’s evaluation and the estimated mammary gland content ratio by regression DCNN.

Figure 4 shows a graph with the mammary gland content ratio evaluated by the physician on the horizontal axis and the estimated ratio by regression DCNN on the vertical axis. We obtained a high correlation of 0.93 (*p* < 0.01) between the evaluation by the physician and the estimated ratio.

**Figure 5:**
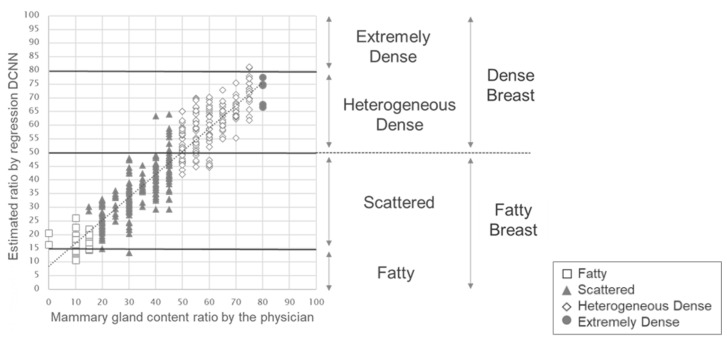
Method for determining breast composition from mammary gland content ratio.

From the estimate of mammary gland content ratio, which yielded a high correlation of 0.93, we searched thresholds of mammary gland content ratio to classify as Fatty, Scattered, Heterogeneous dense, and Extremely dense. We determined thresholds of 15%, 50%, and 80%. These thresholds are shown in Figure 5: less than 15% was defined as Fatty; 15% to less than 50% was defined as Scattered; 50% to less than 80% was defined as Heterogeneous dense, and 80% or more was defined as Extremely dense. These thresholds were also used to categorize the breast composition from the estimated ratio into two types: Fatty and Scattered were grouped as Fatty breast, and Heterogeneous dense and Extremely dense were grouped as Dense breast. Table 2 and Table 3 present the comparisons of the physician’s four categories and the two categories with the estimated ratio by regression DCNN, respectively. The agreement rates were high at 84.8% (kappa = 0.78; 95%CI: 0.73 0.82) in the four categories and 92.0% (kappa = 0.83; 95%CI: 0.77, 0.89) in the two categories, suggesting that breast composition could be estimated using regression DCNN with high accuracy. Importantly, there were no two-step errors or more in the estimated breast composition.

We compared the results of breast composition from the estimated mammary gland content ratio by regression DCNN and the conventional discriminative DCNN method to scrutinize the effectiveness of regression DCNN. The loss function of the network was changed to softmax cross-entropy in discriminative DCNN from regression DCNN. Other parameters remained unchanged. Table 4 compares breast composition by the physician and by discriminative DCNN. The agreement rates were 83.5% (kappa = 0.78; 95%CI: 0.73 0.84) in the four categories (Table 4(a)) and 89.0% (kappa = 0.77; 95%CI: 0.70 0.83) in the two categories (Table 4(b)). However, both results were lower than those of regression DCNN (84.8% (kappa = 0.78), 92.0% (kappa = 0.83)), shown in Table 2 and Table 3. Comparing the estimations by regression DCNN and discriminant DCNN from Table 2 and Table 4a, the agreement rate of regression DCNN was higher than that of discriminant DCNN for Scattered (Table 2 for regression DCNN: 203/400, Table 4(a) for discriminant DCNN: 191/400) and Heterogeneous dense (Table 2 for regression DCNN: 127/400, Table 4(a) for discriminant DCNN: 117/400). The threshold between Scattered and Heterogeneous dense is an indicator of the two categories: Fatty breast and Dense breast. In recent years, it has been debated whether patients with dense breasts need mammography and adjunctive ultrasonography for breast cancer screening [29,30]. However, subjectively evaluating Scattered and Heterogeneous dense [31,32] is challenging. Therefore, high accuracy estimates of Scattered and Heterogeneous dense leads to a highly effective evaluation in daily practice. Moreover, there was a two-step error in the estimated four categories of breast composition by discriminative DCNN (Table 4(a)). Conversely, there were no two-step errors or more in the estimation by regression DCNN (Table 2). Because we used continuous values (the estimated mammary gland content ratio by regression DCNN) to estimate breast composition, two-step errors or more were less likely to occur than with discriminative DCNN estimation.

## 4. Discussion

**Figure 6:**
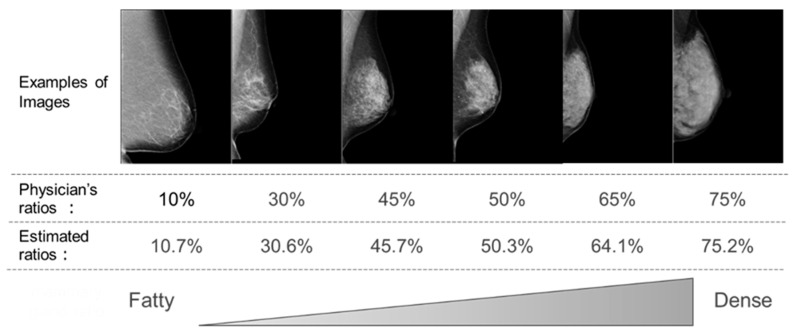
Examples of correctly classified images and results of comparing estimated ratios and visual evaluation.

We developed a regression DCNN to estimate the mammary gland content ratio. Results demonstrate that the mammary gland content ratio can be estimated with high accuracy using regression DCNN. Figure 6 shows six examples arranged by increasing mammary gland content ratio from left to right based on the physician’s evaluation. Comparing mammograms and the estimated ratios in the upper and lower parts of Figure 6, we confirmed that the ratios accurately represent the visual evaluation of mammograms, and the high accuracy with regression DCNN enables their daily usage.

**Figure 7:**
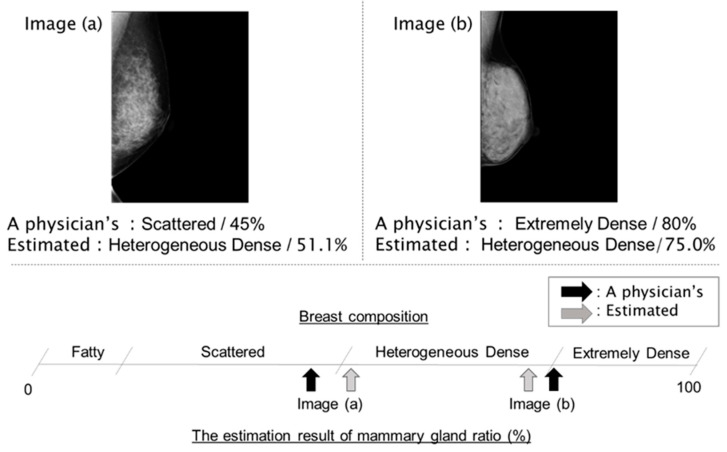
Examples where the four categories of breast composition by the physician and by regression DCNN were not matched.

A total of 61 images of breast composition between the physician’s estimation and the estimation by regression DCNN were not matched. Two examples in these images are shown in Figure 7. Figure 7 (Image a) was categorized as Scattered by the physician but Heterogeneous dense by regression DCNN because the estimated value was 51.1%. Figure 7 (Image b) was classified as Extremely dense by the physician but Heterogeneous dense by regression DCNN because the estimated value was 75.0%. Mammary gland content ratios in both images were different, as shown in Figure 7; however, both were mistaken for Heterogeneous dense. Conversely, the estimated mammary gland content ratio by regression DCNN was 51.1% in Figure 7 (Image a) and 75.0% in Figure 7 (Image b). Therefore, it could be confirmed that the estimated ratio was close to the Scattered and Extremely dense thresholds. For the above 61 images, we calculated the difference between the estimated ratio and the most immediate threshold (15%, 50%, and 80%). Table 5 presents the results summarized by breast composition, classifying the difference as 5% or less, 10% or less, or 15% or less. In all images, the differences were less than 15%. Therefore, by presenting the mammary gland content ratio to the physician concurrently with the classification of breast composition, it is possible to intuitively understand the estimated results according to the estimated ratio using regression DCNN even when the estimated breast composition is incorrect. When a physician is confused about the classification, simply presenting four classifications is not supportive because the physician does not know why the classification was made by AI. In this case, if continuous values are presented, it will be possible to understand that some are Scattered closer to Fatty and others are Heterogeneous closer to Extremely dense; this could be one of the explanations of why the classification was made by AI. In daily practice, we have received positive feedback from physicians regarding the output of continuous values indicating mammary gland content ratio, and we expect that the output of continuous values will be used on a daily basis. When explained the results to the patient or while performing a comparison reading, physicians have to classify the mammograms in the same category even if the mammograms have very different appearances (e.g., the second and third images from the left in Figure 6 are classified in the same category, but they look very different on the mammogram), because there are four categories in common use. This is not sufficient for physicians to explain to patients or decide breast composition. This model could be used to guarantee reliability of these problems. In recent years, Grad-CAM and other explainable AI (XAI, eXplanable AI) for AI outputs have attracted attention, and the method of automatic classification of breast composition from continuous values (mammary gland content ratio) using regression DCNN could be interpreted as a kind of explainable AI, and we believe it will be useful in clinical use.

In this study, the expert physician judged the gold standard (breast composition and mammary gland content ratio) by visual evaluation. In cases where the presence or absence of an abnormal finding is to be determined, it is important to determine the Gold Standard by the consensus of several physicians, because detection errors by physicians could be expected. However, in this study, misclassification errors are less likely to be assumed than when determining the presence or absence of an abnormal finding, so we considered the issue of intervariability from the consensus of several physicians based on subjective evaluation to be more important than the issue of detection error by a single physician. Therefore, we decided to use the judgment results of the expert physician, who has many years of experience and is one of the main members of the guideline development team, as the Gold Standard. However, using this method to determine the gold standard could be a limitation of this research.

## 5. Conclusions

We found that the mammary gland content ratio can be estimated with high accuracy using regression DCNN. The results indicate the effectiveness of regression DCNN and suggest that breast composition could be evaluated with high accuracy from the calculated mammary gland content ratio. Two-step errors or more are unlikely to occur, and the estimated results can be intuitively understood. Therefore, an automated classification of breast composition using this method can be developed to support observer evaluations, which is helpful in medical screening and daily practice.

## Figures and Tables

**Table 1:** Breakdown of the dataset used in this study (training and testing datasets).

Characteristic	Training Set	Test Set
All Images	1076	400
Breast Composition		
Fatty	74	28
Scattered	528	214
Heterogeneous Dense	450	152
Extremely Dense	24	6
System (Screening Period)		
FPD/Cannon (2020/1–2020/8)	598	230
CR/Konica Minolta (2007/11–2008/3)	62	34
FPD/Siemens (2020/11–2021/5)	416	136

**Table 2:** Result of comparing four categories of breast composition by the physician and from the estimated ratio by regression DCNN.

	**Prediction**				
**Truth**		** Fatty **	**Scattered**	**Heterogeneous Dense**	**Extremely Dense**
	**Fatty**	9	19	0	0
	**Scattered**	2	203	9	0
	**Heterogeneous Dense**	0	23	127	2
	**Extremely Dense**	0	0	6	0
				Accuracy: 84.8% (339/400)	

**Table 3:** Result of comparing two categories of breast composition by the physician and from estimated ratio by regression DCNN.

	**Prediction**		
**Truth**		**Fatty** **Breast**	**Dense Breast**
	**Fatty** **Breast**	233	9
	**Dense** **Breast**	23	135
		Accuracy: 92.0% (368/400)	

**Table 4:** Results of breast composition by discriminant DCNN ((a) four categories, (b) two categories).

	**(a) Breast Composition four Categories**						**(b) Breast Composition Two Categories**		
	**Prediction**						**Prediction**		
		**Fatty**	**Scattered**	**Heterogeneous Dense**	**Extremely Dense**			**Fatty** **Breast**	**Dense Breast**
**Truth**	**Fatty**	23	4	1	0	** Truth **	** Fatty ** ** Breast **	233	9
	** Scattered **	15	191	8	0				
	** Heterogeneous Dense **	0	34	117	1		** Dense ** ** Breast **	34	124
	** Extremely Dense **	0	0	3	3				
				Accuracy: 83.5% (334/400)				Accuracy: 89.0% (357/400)	

**Table 5:** Result of differences between the threshold and the estimated mammary gland content ratio summarized by breast composition.

	<5%	<10%	<15%
Fatty	57.9% (11/19)	94.7% (18/19)	100% (19/19)
Scattered	45.5% (5/11)	81.8% (9/11)	100% (11/11)
Heterogeneous Dense	84% (21/25)	100% (25/25)	100% (25/25)
Extremely Dense	16.7% (1/6)	66.7% (4/6)	100% (6/6)
total	62% (38/61)	91.8% (56/61)	100% (61/61)

## Data Availability

The data in this study are available on request from the corresponding author upon reasonable request.
